# Changes in Carbon Pool and Stand Structure of a Native Subtropical Mangrove Forest after Inter-Planting with Exotic Species *Sonneratia apetala*

**DOI:** 10.1371/journal.pone.0091238

**Published:** 2014-03-11

**Authors:** Weizhi Lu, Shengchang Yang, Luzhen Chen, Wenqing Wang, Xiaona Du, Canmou Wang, Yan Ma, Guangxuan Lin, Guanghui Lin

**Affiliations:** 1 Key Laboratory of the Ministry of Education for Coastal and Wetland Ecosystems, School of Life Sciences, Xiamen University, Xiamen, Fujian, China; 2 Division of Marine Sciences and Technology, Graduate School at Shenzhen, Tsinghua University, Shenzhen, Guangdong, China; 3 Administrative Bureau of Zhanjiang National Mangrove Nature Reserve, Zhanjiang, Guangdong, China; 4 Ministry of Education Key Laboratory for Earth System Modeling, Center for Earth System Science, Tsinghua University, Beijing, China; Dauphin Island Sea Lab, United States of America

## Abstract

In this study, we compared stand structure, biomass and soil carbon pools, and litterfall production between a mixed mangrove forest consisting of *Aegiceras corniculatum* inter-planted with the exotic *Sonneratia apetala* and a native monospecific forest dominated by *A. corniculatum* in the intertidal area of Zhanjiang, Guangdong Province, southeast China. The goal of this study was to test the hypothesis that inter-planting fast growing exotic mangrove *S. apetala* into subtropical native mangrove forests will significantly increase C sequestration. Although the tree heights and basal diameters of *S. apetala* were significantly higher than those of *A. corniculatum*, the density of the 12-year-old *S. apetala* trees in the mixed forest was much smaller than that of *A. corniculatum* in the monospecific forest. In contrast to several previous studies on *S. apetala* forests planted directly on mangrove-free mudflats, the mixed mangrove forest showed no significant difference in either standing biomass or soil carbon pools from the native monospecific mangrove forest (*p* = 0.294 and 0.073, respectively) twelve years after inter-planting with *S. apetala*. Moreover, carbon cycling was likely speeded up after inter-planting *S. apetala* due to higher litterfall input and lower C/N ratio. Thus, inter-planting fast-growing *S. apetala* into native mangrove forest is not an effective way to increase carbon sequestration in this subtropical mangrove forest. Given that exotic plant species may exert negative impact on native mangrove species and related epifauna, this fast-growing mangrove species is not suitable for mangrove plantation projects aiming mainly at enhancing carbon sequestration.

## Introduction

Mangrove wetlands have great ecological and economic value, including high primary productivity, effective carbon (C) storage, high epifaunal diversity and great benefits for aquaculture [1]–[5]. However, there is increasing concern about the continued loss and degradation of mangroves, as 35% to 86% of the global mangrove area has been lost during the last several decades [6]–[9]. In addition, as international climate agreements emphasize Reduced Emissions from Deforestation and Degradation (REDD+) as a key option for mitigating climate change [10], attention has been drawn to C sequestration potentials of mangrove forests, salt marshes and seagrass beds, as well as measures to enhance these C sinks [10]–[12].

Afforestation and reforestation can be effective methods for increasing forest ecosystem C sequestration [13], [14]. Large-scale efforts have been made to restore degraded mangroves and create new mangrove forests around the world [15], [16]. In the tropical and subtropical areas of mainland China, plantation is a common method for restoring mangrove forests [17]. Native to Bangladesh, *Sonneratia apetala* Buch. Ham, a fast-growing mangrove tree species of Sonneratiaceae family, has been widely used for mangrove restoration projects in many locations along the southeastern coasts of China during last three decades. It was estimated that the total area of *S. apetala* plantations in China reached 3800 ha, which accounts for more than 50% of total replanted mangrove area [17]. However, there were still debates on whether *S. apetala* in China is an invasive species or a great restoration species [18].

In most cases, *S. apetala* afforestation projects were implemented on mudflats with low salinity, where native mangrove forests had been removed or destroyed [18]. In Guangdong and Fujian provinces, however, *S. apetala* trees were often inter-planted inside native mangrove forests for landscape renovation to make the appearance prettier because of *S. apetala* tall and “willow-like” canopy. Moreover, this exotic mangrove species has been naturally spread into many native mangrove forests in these regions since it was introduced to China in late 1970’s [18]. Previous researches have studied carbon accumulation potentials of *S. apetala* plantations in destroyed mangrove forests. For example, Ren et al. demonstrated that *S. apetala* plantations sequestered large amounts of C in both biomass and sediments [19]. Other studies also showed that *S. apetala* has higher rates of C accumulation in biomass and sediments than many native mangrove species [19]–[22]. However, these previous studies focused only on monoculture plantations of *S. apetala* on mudflats. Little was known about whether inter-planting *S. apetala* into native mangrove forests can also increase C accumulation. In this study, we hypothesized that planting this exotic fast-growing mangrove species into native mangrove forests should have greater potentials to increase C accumulation than native mangroves.

In the present study, we compared stand structure, C pools in standing biomass and sediments, and litterfall production between a subtropical mangrove forest dominated by native *Aegiceras corniculatum* (L.) Blanco and a mixed mangrove forest of *A. corniculatum* inter-planted with the exotic *S. apetala*. The objective of this study was to test the hypothesis that inter-planting fast growing exotic mangrove species such as *S. apetala* into subtropical mangrove forests will significantly increase C accumulation. *A. corniculatum* of the Myrsine family, commonly known as black mangrove, is a species of mangrove shrub with multiple stems, which has a large distribution area in southeast China.

## Materials and Methods

### Study site description

**Figure 1:**
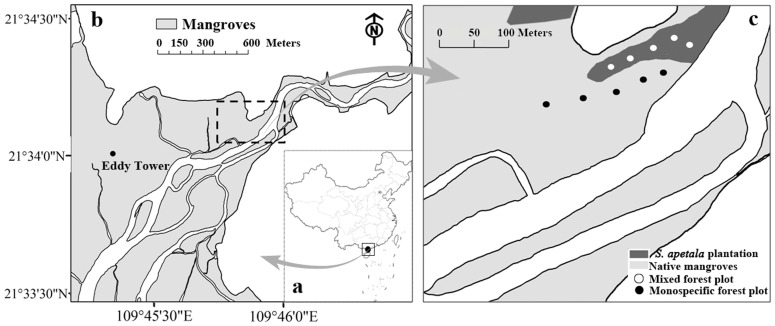
The location of Gaoqiao mangrove research station (a and b) and the experimental plots in the mixed and monospecific mangrove forests (c).

The study site (21.5676°N, 109.7562°E) is located at the confluence of Liangguang River and Gaoqiao River, northeast of the Beibu Gulf, in Guangdong Province. The Gaoqiao mangrove forest is at the core zone of Zhanjiang Mangrove National Nature Reserve (Fig. 1), the largest mangrove nature reserve in China with a total mangrove area of approximately 1200 ha.

**Figure 2:**
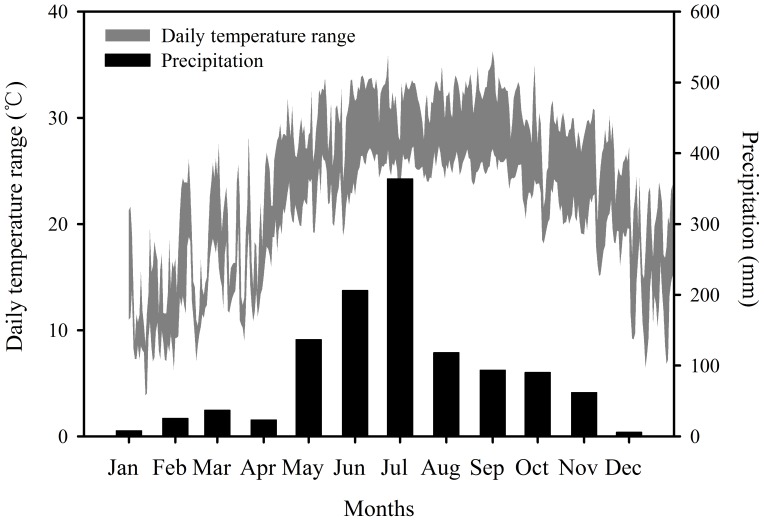
Daily air temperature range and monthly precipitation in 2010 for the Gaoqiao mangrove research station.

The local climate is characterized by strong seasonal variation with high precipitation. According to the data from our eddy flux tower (about1.0 km apart from the study site) (Fig. 1), the annual precipitation at our study site was 1168 mm in 2010, and the air temperature ranged from 8.7°C to 37.3°C, with an annual mean of 23.2°C (Fig. 2). The study site experiences regular diurnal tides, with an inundation once during each lunar day, and the average tide range is 1.3 m but the highest astronomical tides can reach up to 1.9 m above the soil surface.

The dominant mangrove species at the study site was *A.corniculatum*, accompanying by *Bruguiera gymnorrhiza* (L.) Lamk, *Rhizophora stylosa* Griff and *Kandelia obovata* Sheue, Liu et Yong, forming separate pure patches in the mixed forest. Most *A. corniculatum* trees were in dwarf form, with a mean canopy height of about 2.2 m. The age of these mangrove trees was estimated to be greater than 60 years. In 1999, the seedlings of *S. apetala* were planted into a monospecific *A. corniculatum* tree stand of about 1 ha and then developed gradually into a mixed forest. The initial seedling density of *S. apetala* was set at 1,667 seedlings ha^−1^ and the average row width was approximately 1 m. At the time of inter-planting, the mean height of *S. apetala* seedlings was about 40 cm (personal observation by Guangxuan Lin, one of the co-authors of this paper and the staff at Zhanjiang National Mangrove Nature Reserve).

### Forest structure surveys and biomass measurements

We investigated two mangrove forests for this study after applying for the permission of the Zhanjiang Mangrove National Nature Reserve. The first forest was a native mangrove forest (hereafter ‘monospecific forest’), composed of only ∼60 year old *A. corniculatum* trees. The other was a mixed forest, composed of 12 year old *S. apetala* trees inter-planted into∼60 year old *A. corniculatum* forest (hereafter ‘mixed forest’). Five 10 m ×10 m survey plots (as replicates for each forest type) were selected along the middle line of each forest at 50 m intervals (Fig. 1).

In January 2011, we recorded canopy height (*H*) and diameter at breast height (*DBH* or *D* in this paper) of each *S. apetala* individual tree in all five quadrats as mentioned above. Because of their extensive root systems, excavating trees of *S. apetala* would have been very destructive to surrounding trees. Instead, the relative growth equations obtained from a similar *S. apetala* forest in northwest Leizhou Bay, about 75 km south the study site [19], were used to estimate the aboveground biomass (*W*_AGB_), belowground biomass (*W*_BGB_) and total biomass (*W*_Total_) using their *D* and *H* values:
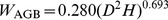
(1)

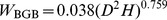
(2)

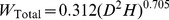
(3)


One shortcoming of this approach for *W*_BGB_ measurements was that the aerial roots were not separated from normal roots [19], even though they functioned differently and might have very different carbon content.

We also surveyed all trees of *A. corniculatum* in both the mono-specific native mangrove forest and the mixed mangrove forest, and recorded the tree heights. Although the stems of *A. corniculatum* trees were usually higher than 1.3 m, most of them had more than one branch at the height of 1.3 m. Thus, we measured the basal diameters and basal areas at the height of 5 cm above the soil surface for *A. corniculatum* forest structure survey. We didn’t use relative growth equations to estimate the biomass of *A. corniculatum* because we had no suitable allometric growth equations for shrub mangrove species with multiple stems. Instead, three1 m ×1 m quadrats were selected in the center of each forest for the measurements of *A. corniculatum’s* aboveground biomass (AGB) and belowground biomass (BGB). The AGB included the total weight of leaves, branches, stems and trunk, but the biomass of the propagules (semi-viviparous fruits) was excluded since it was only a very small proportion of total biomass, although the propagules could be an important component of litterfall during the reproductive months (see below). For the BGB measurements, the soils within a soil cube of 1 m^3^ were excavated, and then carefully washed over a fine screen to collect all the roots. For the mixed mangrove forest, the roots of *S. apetala* were removed by hands because their biomass was already estimated by the allometric growth equations mentioned above. These roots had more spongy cortex than those of *A. corniculatum*, facilitating their identification between each other. The fresh weights of AGB and BGB components from each quadrat were measured in the field using a balance (MS24KLIPE, Metteler-Toledo, Greifensee, Switzerland). The biomass of each component was then calculated using the above data.

### Biomass C/N ratio and carbon pool measurements

About 100–200 g fresh weight of each biomass component (leaves, branches and roots) from five trees of each species in the two forest-types were sub-sampled for water, carbon (C) and nitrogen (N) content analyses. We used the mean value of five trees in the same survey plot for each forest type as one replicate, so there were only five replicates for each species or forest type. These samples were oven dry at 60°C for at least 72hours to a constant weight, and then their dry weights were measured for water content calculations. The dried samples were tested for C and N contents using an elemental analyzer (Vario EL III Elemental Analyzer, Hanau, Germany). The C storages in all biomass components of two mangrove species were calculated separately using their biomass and C content data, and then summed up for both the monospecific and mixed mangrove forest.

### Litterfall collection

Litterfall was collected using 1 m^2^ round baskets covered with 1.5 mm fiberglass mesh. One basket was set up in each plot, with a total of 10 baskets for the two mangrove forests. The baskets were placed 1.5 m above the soil to avoid immersion by tidal water, and litterfall was collected twice a month from January 1, 2010 to December 31, 2010. Leaves, branches, twigs and fruits (or propagules, i.e. semi-viviparous fruits, for *A. corniculatum*) were separated manually and dry weight was determined using an electronic balance (ML2001, Metteler-Toledo, Greifensee, Switzerland) after oven-dry at 60°C for at least 72 hours to a constant weight. The components of litterfall were not separated between two species in the mixed mangrove forest due to the labor shortage in the field. Monthly litterfall production was calculated using the accumulated all litter during each month.

### Soil sampling and property measurements

Soil samples down to 60 cm depth were collected using a soil core sword (C040903, Eijkelkamp, Holland) in July 2010. Five cores were randomly taken in each plot, for a total of 50 cores collected for the two mangrove forests. Each core was cut into three sections: 0–20 cm, 20–40 cm and 40–60 cm. Fixed-volume cores were also taken in each section with a soil-cutting ring to measure bulk density. These soil samples were dried at 60°C for at least 72 hours to a constant weight and the dry weights were taken using the Metteler-Toledo’s ML 2001 electronic balance. Soil bulk density was calculated as the ratio of soil dry weight to the volume of the soil-cutting ring. The dried samples were ground with a ball grinding mill (Retsch Mixer Mill MM400, Verder, Germany) and passed through an 80-mesh sieve. A 10-mg subsample was taken from each soil sample and then measured for its C and N contents as described above.

### Statistical analyses

For each parameter, we calculated the mean and standard deviation (SD) of the five replicates for each forest. The differences in tree density, basal area, height, biomass, C/N ratio among forest types were evaluated using one-way ANOVA test. The assumptions for the one-way ANOVA test, including normal distribution and homogenous variance of the data, were first tested. Since the tree density, basal area, height and leaf C/N ratio had an inhomogeneous variance, we log-transformed these values before the ANOVA tests. Repeated measure ANOVA was used to examine the seasonal change in litterfall production over time in two mangrove forests. The differences in the biomass and soil C pools between the two forest types were evaluated by *t*-test after a power analysis was conducted. All statistical analyses were performed using SPSS version 13.0 (SPSS Inc., Chicago, USA). Power analysis was performed using PASS software version 2008 (Kaysville, Utah, USA).

## Results

### Forest structure and biomass

The tree height of the 12 year-old *S. apetala* was significantly higher (*p*<0.001) than *A. corniculatum* (Table 1). Both basal area and tree height of *A. corniculatum* in the mixed forest were significantly lower (both *p*<0.001) than those in the monospecific forest (Table 1). Moreover, the basal area and tree height of *S. apetala* were significantly higher (*p*<0.001) than those of *A. corniculatum* in the same forest (Table 1). There was no significant difference (*p* = 0.425) in the tree density of *A. corniculatum* between the mixed and monospecific forests, which was about 30 times greater than that of *S. apetala* in the mixed forest (Table 1).

**Table 1:** Selected properties of soils and tree canopy structure for two different mangrove forests (mean±SD).

Forest type	Soil texture	Species	Age (yrs)	Height (m)	Basal diameter (cm)	Basal area (m^2^ ha^−1^)	Density (ind ha^−1^)
Mixed forest	Silty clay	*S. apetala*	12	13.64±0.88^c^	15.86±3.31^c^	73.15±9.80^b^	1866.67±568.62^a^
		*A. corniculatum*	∼60	1.74±0.08^a^	2.94±0.16^a^	38.48±5.43^a^	56846.67±10826.27^b^
Monospecific forest	Silty clay	*A. corniculatum*	∼60	2.02±0.05^b^	4.02±1.25^b^	80.82±12.47^b^	63200±6053.10^b^
		*F*_2,6_		1813.27	58.01	16.29	66.40
		*p* value		<0.001	<0.001	<0.001	<0.001

Different lower case letters indicate significant differences at *p*<0.05 between two mangrove species or the same species between two forest types.

The total amounts of biomass in the monospecific and mixed forests were comparable at 102.54±9.83 and 126.33±32.19 Mg ha^−1^, respectively (*t*-test, *p* = 0.280)(Table 2). However, the total biomass of *A. corniculatum* in the monospecific forest was four times higher than that in the mixed forest (Table 2). The 12-year-old *S. apetala* comprised the majority of the biomass in the mixed forest (about 77.13%) (Table 2). The biomass components of *S. apetala* had significantly lower C/N ratios than their counterparts of *A. corniculatum* except the branches (*p* = 0.084, 0.030 and 0.020 for the branches, leaves and roots, respectively) (Table 3). However, the C/N ratios of *A. corniculatum* biomass were not significantly different between the mixed and monospecific mangrove forests (*p* = 0.689, 0.439 and 0.379 for the branches, leaves and roots, respectively).

**Table 2:** Aboveground biomass (AGB) and belowground biomass (BGB) of two different mangrove forests (mean±SD) (Unit: Mg ha^−1^)*.

Forest type	Species	Stem or Trunk	Branch	Leaf	AGB	BGB	Total
Mixed forest	*S. apetala*	49.27±15.54^b^	21.58±4.87^b^	4.39±0.54^a^	79.28±21.39^b^	18.47±5.42^b^	97.45±26.72^b^
		(50.12±2.39%)	(22.36±1.25%)	(4.57±0.32%)	(81.42±0.41%)	(18.87±0.43%)	
	*A. corniculatum*	11.67±4.28^a^	2.84±0.86^a^	2.56±0.37^a^	17.07±5.47^a^	11.81±0.65^a^	28.88±5.47^a^
		(39.37±8.27%)	(9.68±1.25%)	(8.94±0.60%)	(57.99±8.83%)	(42.04±9.28%)	
Monospecific forest	*A. corniculatum*	58.75±5.94^b^	14.85±2.71^b^	9.58±1.20^b^	83.17±9.83^b^	19.37±2.55^b^	102.54±9.83^b^
		(57.27±0.41%)	(14.40±1.32%)	(9.31±0.30%)	(80.99±1.86%)	(19.17±4.37%)	
*F*_2,6_		18.91	25.54	48.50	21.19	4.232	18.17
*p* value		0.003	0.001	<0.001	0.002	0.071	0.003

*Here AGB did not include the biomass of *A. corniculatum* propagules (semi-viviparous fruits), while the BGB of *S. apetala* included all aerial roots. Different lowercase letters indicate significant differences at *p*<0.05 between two mangrove species or the same species between two forest types. The values in the parentheses were the percentage of each component biomass to the total biomass for a given species.

**Table 3:** C/N ratios of soils and plant biomass for two types of mangrove forests (mean±SD).

Forest type	Soil	Species	Branch	Leaf	Root
Mixed forest	17.04±1.23^a^	*S. apetala*	96.47±14.37^a^	31.15±2.58^a^	163.3±23.71^a^
		*A. corniculatum*	197.06±22.75^a^	53.01±7.73^b^	395.06±65.70^b^
Monospecific forest	20.71±2.75^b^	*A. corniculatum*	177.13±76.80^a^	49.15±0.83^b^	324.86±104.19^ab^
		*F*_2,6_	3.86	26.53	8.08
*t*_4_	–4.76	*p* value	0.084	0.030	0.020
*p* value	0.001				

Different lowercase letters indicate significant differences at *p*<0.05 between two mangrove species or the same mangrove species between forest types.

### Litterfall production

**Figure 3:**
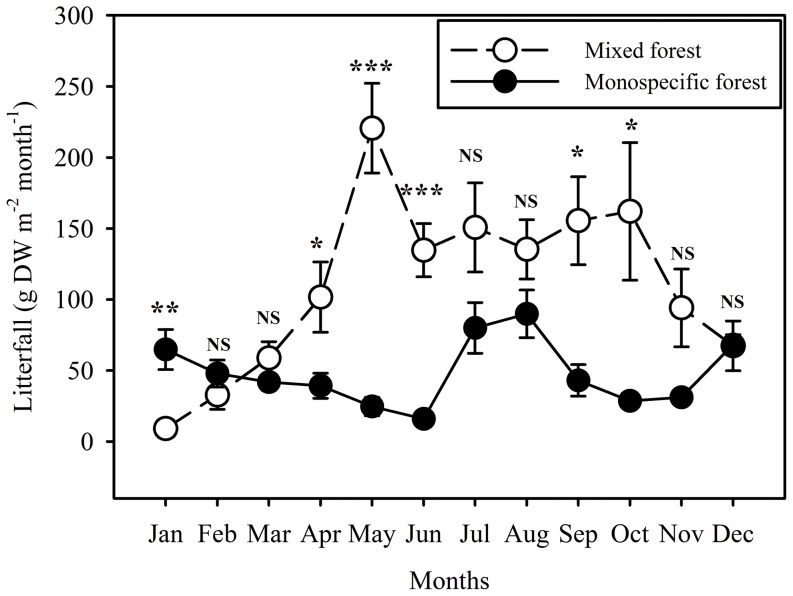
Monthly litterfall production for the *A. corniculatum* monospecific mangrove forest and the mixed mangrove forest of *S. apetala+A. corniculatum* from January to December 2010 (mean±SD; n = 5). Significant differences at each month between two forests are indicated by *p<0.05, **p<0.01 and ***p<0.05, while NS indicates not significant difference at *p*>0.05. Note that in the mix forest the litter from two mangrove species were not separated.

Annual litterfall production during 2010 was 1322.5 and 574.7 g m^−2^ for the mixed and monospecific forest, respectively (Fig. 3). Litterfall from the mixed forest showed a significant seasonal dynamic (*p*<0.001), with a gradual increase from January, reaching a peak in May and then maintaining relatively high production from May to October (Fig. 3). The two peak litterfall production periods were May and October (Fig. 3). However, litterfall production of *A. corniculatum* did not show obvious seasonal pattern except for a peak production in July-August (Fig. 3). Moreover, there were significant differences in the litterfall production between two forests over months of the year (repeated measure ANOVA, *p<*0.001).

### Soil properties

**Figure 4:**
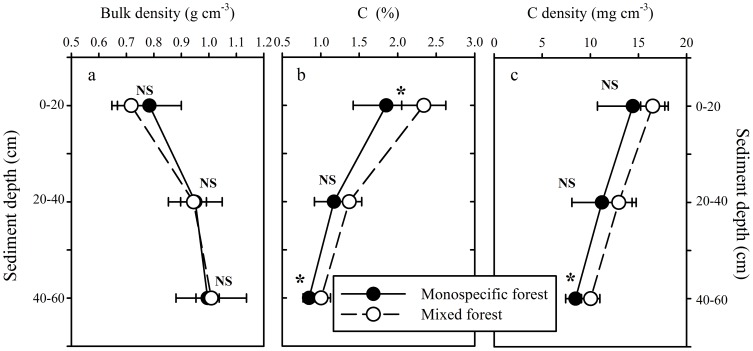
Bulk density (a), carbon concentration (b), and carbon density (c) at different soil depths for a mixed mangrove forest and a monospecific mangrove forest (mean±SD; n = 5). Significant differences between two forests are indicated by *p< 0.05, **p<0.01 and ***p<0.05, while NS indicates no significant difference at *p*>0.05.

There was no significant difference in bulk density differ between two forests at each depth (Fig. 4a). However, the soil C concentration in the mixed forest was significantly higher than in the monospecific forest at both 0–20 cm and 40–60 cm layers (Fig. 4b). There was no significant difference in C density between the two forest types at both 0–20 cm and 20–40 cm layers (Fig. 4c).The C/N ratio of top layer soils in the mixed mangrove forest was significantly lower (*p*<0.001) than that in the monospecific forest (Table 3).

### Carbon storage in biomass and soil

**Figure 5:**
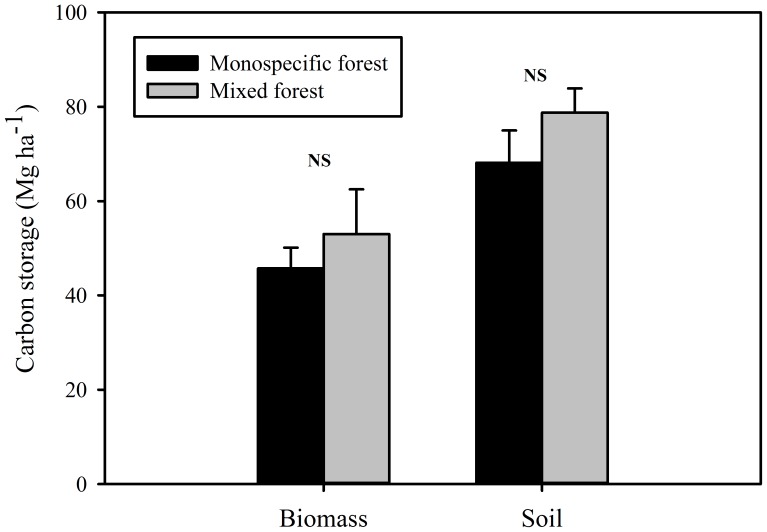
Comparison in the carbon storage of biomass and soils between a mixed mangrove forest and a monospecific mangrove forest. Significant differences between two forests are indicated by *p< 0.05, **p<0.01, ***p<0.05, while NS indicates not significant difference at *p*>0.05.

Total C storage in biomass was 45.73±4.43 Mg C ha^−1^ and 53.03±9.49 Mg C ha^−1^ for the monospecific forest and mixed forests, respectively, and the total C storage in soil was 68.15±6.81 and 78.76±5.13 Mg C ha^−1^, respectively (Fig. 5). However, there was no significant difference in biomass C storage between the two mangrove forests (*t*-test, *p* = 0.294; with a result of 0.805 from the power analysis) (Fig. 5). Furthermore, there was no significant difference in soil C storage between the two mangrove forests (*t*-test, *p* = 0.073; with a result of 0.761 from the power analysis) (Fig. 5).

## Discussion

### C sequestration potential of *S. apetala* forests

Compared with native mangrove species such as *A. corniculatum*, *Avicennia marina*, *K. obovata* and *B. gymnorrhiza*, the exotic mangrove species *S. apetala* has much greater biomass amount and C accumulation capacity [19]–[23]. For example, Ren et al. observed that the total C stock in a 10-year-old *S. apetala* plantation in Leizhou, Guangdong was 49.0 Mg C ha^−1^ [19], while Liao et al. reported that the total aboveground biomass in a 10-year-old *S. apetala* plantation in Haikou, Hainan reached 82.1 Mg DW ha^−1^ [20]. Chen et al. also demonstrated experimentally that at least during the first 25 months the total C stock in *S. apetala* planting in Shenzhen, Guangdong was up to 18.74 Mg C ha^−1^ [24]. Because of such high C sequestration capacity, this fast-growing exotic mangrove species from Bangladesh is often recommended for coastal afforestation or restoration projects aiming at increasing C sink [19], [22], even though it potentially becomes an invasive species in the southern coast region of China and posses serious threats to native mangrove forests [18].

In contrast to these above studies where the *S. apetala* trees or seedlings were planted directly on mangrove-free mudflats, the C storage did not increase 12 years after the *S. apetala* seedlings was inter-planted into the native mangrove forest of *A. corniculatum* forest in the present study. Although the *S. apetala* trees were significantly taller and bigger than the native mangrove species *A. corniculatum*, the C storage in the biomass of the mixed forest was only slightly increased but not significant when compared to the native monospecific mangrove forest of *A. corniculatum*. We found that the biomass of the native mangrove *A. corniculatum* in the mixed forest was just a quarter of the same species’ biomass in the monospecific forest due to significantly smaller basal diameter, lower basal area and lower tree height. Moreover, the density of the 12-year-old *S. apetala* trees was just about a thirtieth of that for the *A. corniculatum* trees in the monospecific forest. The total C storage in the mixed forest did not show significant increase after inter-planting *S. apetala* trees because the increase in biomass C accumulation by *S. apetala* was almost compensated by the decrease in plant biomass of *A. corniculatum* trees underneath them.

Most previous studies focused on C accumulation in biomass following restoration, neglecting C accumulation in soils, which could be very important in determining long-term C sequestration [5], [25]. Our results showed that C accumulation in the soil in these subtropical mangrove forests was greater than that in the biomass, which was consistent with a previous study [10]. A previous study showed that *S. apetala* monoculture plantations could sequestrate significant amounts of C in sediments probably due to the increasing input of dead roots and litterfall [19]. However, Chen et al. found no significant increase in soil carbon-storage in *S. apetala* monoculture forests [24]. The study of Chen et al. only measured the C accumulation in the top 20 cm soils and the experiment lasted less than 3 years after the seedlings were planted, which could substantially underestimated the total C storage in the sediments. In the present study, we investigated the C pools in the sediments down to 60 cm deep and focused on *S. apetala* trees of 12 years old, but we still did not find significant increase in C storage in the sediment after inter-planting *S. apetala* into existing native mangrove forest. Thus, it will take much longer time for the exotic mangrove plants to increase C accumulation in sediments through the increasing input of dead roots and litterfall.

### Change in C turnover following introduction of exotic mangrove species

Previous studies demonstrated that, following the invasion of alien species, ecosystems may become more fragile and unstable due to unstable C pools and fluxes [26], [27]. In most cases, litterfall production was significantly higher in the alien forest than native forests [28]–[30]. This phenomenon was confirmed by the comparison in the litterfall production between alien *S. apetala* and native mangrove species in China [31]. Zan et al. observed an annual litterfall production of 1660 g DW m^−2^ for a *S. apetala* forest in Shenzhen [31], while Han et al. reported even higher litterfall production (1895 g DW m^−2^ ) in a six- to seven-year-old forest of *S. apetala* in Leizhou, Guangdong [32]. The results for the litterfall production of *S. apetala* forest from the present study were comparable with these values, but much greater than those of many native mangrove forests in the same region (average annual litterfall production was only 1049±295 g DW m^−2^ ) [33]–[38]. Thus, our results indicated that inter-planting *S. apetala* seedlings into native mangrove forest could supply significantly higher amount of litterfall to mangrove soils, which would speed up the C cycling. In addition, C and N interactions play a key role in determining the speed of C decomposition and thus C loss, and lower C/N ratio is often associated with faster litter decomposition [39], [40]. In the present study, we found that *S. apetala* tissues had significantly lower C/N ratio than those of *A. corniculatum*, with the exception of the branch.

From significantly higher litterfall production and lower biomass C/N ratio associated with the exotic mangrove species *S. apetala*, we can conclude that inter-planting *S. apetala* trees into native mangrove forest can substantially increase the speed of C cycling, which may lead to C loss from the mangrove forests.

It is worthy pointing out that the leaf biomass in the monospecific stand was higher than in the mixed stand (native and non-native together). There are three reasons for this phenomenon. First, the litterfall in the mixed forest composed of more branches and fruits mainly from *S. apetala* than the mono-specific forest. Secondly, the life-span of *A. corniculatum* leaves was more than 24 months, much longer than that of *S. apetala* leaves (about 6.5 months) [41], [42].Thirdly, the biomass allocation in *A. corniculatum* changed significantly after the *S. apetala* inter-planting, with 80.99±1.86% of total biomass allocated to aboveground biomass in the monospecific native mangrove forest but only 57.99±8.83% to the same biomass component in the mixed forest (Table 2). The reduced allocation to AGB after *S. apetala* inter-planting came from all biomass components, with the stem (trunk) biomass decreased the most (Table 2).

### Implications for managements of mangrove restoration projects

Mangrove forests are recognized among the most productive and C rich ecosystems in the world, having critical ecological resources and providing great ecosystem services to human beings [5], [11], [43]. However, mangrove forests are being destroyed at an average annual rate of 1–2% [1], and the situation is much worse in China, with almost half of national mangrove forests being destroyed or degraded [17]. Since the 1990s, the Chinese government has invested greatly to reforest and afforest mangroves to increase the coastal vegetation cover, to protect the shoreline from tidal surges, and to conserve biodiversity along the south-eastern coastlines of China [17], [18].

The planting of *S. apetala* is a core approach to restore mangrove wetlands and thus increase coastal C sequestration in China [17], [19]. However, when *S. apetala* trees are inter-planted into native mangrove forests, interspecific competition is not taken into consideration in most of the reforestation project [24]. Our study found that *S. apetala* had a largely negative effect on native mangroves such as *A. corniculatum* possibly because of shading effects. After the exotic *Sonneratia* trees were planted in the native mangrove forest, lots of pre-existing trees died off because of shading effects. The fast-growing *Sonneratia* trees quickly grew over the existing *Aegiceras* tree canopy shading the pre-existing trees, and then new seedlings of *Aegiceras* grew under the canopy of *Sonneratia* trees, which might have caused them having smaller basal diameters than the trees in the nearby intact native mangrove forest. Unfortunately there was no data collection before the *Sonneratia* planting to verify this process, but it was quite possible according to the field observations by Guangxuan Lin. In a separate study on the same sites of this study, Cai et al. found that there were quite different species composition, animal density and secondary productivity between the *S. apetala* and *A. corniculatum* forests [44]. Thus, more studies are needed to understand the overall impact of alien species on native plant and animal communities of mangrove forests. Given that exotic *S. apetala* may exert negative impact on native mangrove species with only marginal increases in C sequestration, we would not recommend this fast-growing exotic mangrove species for mangrove reforestation or afforestation projects aiming at enhancing C sequestration.
